# Diagnostic Accuracy of Serum and Urine S100A8/A9 and Serum Amyloid A in Probable Acute Abdominal Pain at Emergency Department

**DOI:** 10.1155/2018/6457347

**Published:** 2018-07-03

**Authors:** Arash Forouzan, Kambiz Masoumi, Fakher Rahim, Meisam Moezzi, Ali Khavanin, Nastaran Ranjbari, Malehi Amal Saki, Amirali Fallah Amoli, Niloofar Akhiani, Fatemeh Ghourchian

**Affiliations:** ^1^Department of Emergency Medicine, Imam Khomeini General Hospital, Ahvaz Jundishapur University of Medical Sciences, Ahvaz, Iran; ^2^Health Research Institute, Research Center of Thalassemia & Hemoglobinopathy, Ahvaz Jundishapur University of Medical Sciences, Ahvaz, Iran; ^3^Metabolomics and Genomics Research Center, Endocrinology and Metabolism Molecular - Cellular Sciences Institute, Tehran University of Medical Sciences, Tehran, Iran; ^4^Department of Pathology, Imam Khomeini General Hospital, Ahvaz Jundishapur University of Medical Sciences, Ahvaz, Iran; ^5^Department of Biostatics and Epidemiology, School of Public Health, Ahvaz Jundishapur University of Medical Sciences, Ahvaz, Iran

## Abstract

**Study Design:**

This study was performed to investigate the diagnostic values of some inflammatory biomarkers in abdominal pain.

**Methods:**

Patients over 18 years of age with acute recent abdominal pain who presented to the Emergency Department were evaluated. Serum and urinary samples were taken and evaluated for serum and urine S100A8/A9 and serum amyloid A. All patients were referred to a surgeon and were followed up until the final diagnosis. In the end, the final diagnosis was compared with the levels of biomarkers.

**Results:**

Of a total of 181 patients, 71 underwent surgery and 110 patients did not need surgery after they were clinically diagnosed. Mean levels of serum and urine S100A8/A9 had a significant difference between two groups, but serum amyloid A did not show. The diagnostic accuracy of serum S100A8/A9, urine S100A8/A9, and serum amyloid A was 86%, 79%, and 50%, respectively, in anticipation of the need or no need for surgery in acute abdominal pain.

**Conclusions:**

Our study showed that in acute abdominal pain, serum and urine S100A8/A9 can be useful indicators of the need for surgery, but serum amyloid A had a low and nonsignificant diagnostic accuracy.

## 1. Introduction

Abdominal pain is one of the most common complaints that patients present to the hospital, making up 10% of all cases referred to the emergency department (ED) [1, 2]. Many patients have nonspecific abdominal pains that do not aid in diagnosing a specific disease and represent mild and self-limiting conditions to life-threatening diseases [3, 4]. Nontraumatic acute abdominal pain is a medical emergency that lasts up to 5 days and does not result from trauma [5].

Acute abdominal pain is the most common cause of emergent surgery and one of the most frequent causes for referral to the ED as well as for counseling and admission of nontraumatic patients [6]. The most prevalent causes of emergent abdominal pain that need intervention within 24 hours include acute appendicitis, acute diverticulitis, acute cholecystitis, and bowel obstruction. The most prevalent causes of nonemergent cases include nonspecific abdominal pain and gastrointestinal diseases [1, 4].

Abdominal pain has a vague quality, because of the convergence of the second neuron of organs and abdominal structures in the posterior column of the spine. However, physical examination may reveal different findings in the patients and the location and the severity of pain may change over time, which makes the diagnosis of the causative agent a challenge for the physician [1, 7].

On time diagnosis plays a key role in deciding the treatment process of the patients, while delayed intervention leads to more complications, such as peritonitis and perforation, associated with higher rates of morbidity and mortality [3, 4, 8, 9].

Finding the causes of abdominal pain can be facilitated by imaging tools and methods. Ultrasonography, with medium sensitivity and specificity, can be used to diagnose the underlying disease, but it requires experienced and efficient operators, who may not always be available at health care facilities [10]. In the present times, the increasing use of computed tomography (CT) scans is often not required as the patient rarely gains any advantage and is exposed to harmful radiation and contrast nephropathy and has to bear additional costs [7, 11]. However, despite the use of imaging methods, the diagnostic dilemma still remains challenging as it includes wrong diagnosis or unnecessary surgery [4, 5].

The use of new inflammatory biomarkers is a noninvasive and harmless method [12]. Some biomarkers can help find a proper diagnosis and reduce the use of imaging techniques, if they can be measured in serum, plasma, urine, or feces of the patients, quickly, and are accompanied by high sensitivity, reliability, and low costs [3, 13, 14]. Laboratory, animal, and human studies have shown the feasibility of serum and urine S100A8/A9 and serum amyloid A in inflammatory conditions [3, 7, 13, 15–19].

S100A8/A9 is a complex of the proteins S100A8 and S100A9 in mammals [20, 21]. It has a high tendency to calcium, zinc, and manganese [22–24]. S100A8/A9 was introduced in 1980, for the first time, as an antimicrobial protein that destroys zinc in mammals [20, 21, 25]. Now, it has been found that its antibacterial and antifungal properties are due to its ability to destroy manganese [25]. This protein includes up to 60% of soluble proteins in neutrophil cytosols and, to a lesser extent, in monocytes; it includes macrophages and squamous epithelial cells [21–23]. Mammals secrete this protein during the inflammatory response. The exact mechanism for its secretion is unknown [26].

Serum amyloid A, a protein of HDL, is associated with the apolipoprotein family in plasma. It is mainly produced in the liver [27]. Some actions of these proteins include transferring cholesterol into the liver, calling immune cells to the site of inflammation, and inducing enzymes that break the extracellular matrix down. Small inflammatory responses can also increase the production of serum amyloid A. Plasma levels of serum amyloid A increase with injuries, infections, and inflammatory diseases, such as rheumatoid arthritis and Kawasaki, as well as with viral and autoimmune diseases and malignancies. It is an acute-phase reactant, increasing over a few hours after the inflammatory stimulus, which may increase even more than the C-reactive protein (CRP) [1, 9, 28, 29].

Although clinical findings and conventional biomarkers play a major role in the diagnosis of acute appendicitis or lower abdominal pain etiology, several studies suggest that S100A8/A9 in serum and in the intestine, alone or in combination with other biomarkers, can be a useful accessory and tool for clinical findings [7–30]. In other studies, using stool S100A8/A9 has been approved as a noninvasive method to differentiate the inflammatory versus noninflammatory causes of gastrointestinal diseases [13, 31–36]. Furthermore, Muhammad et al. reported that the diagnostic accuracy of serum amyloid A biomarker for acute appendicitis is higher than that of CRP [19].

Despite the technological progress in accuracy, rapidity, and the ease of diagnosing methods for abdominal pain, many challenges still remain to be addressed. Thus, an accessory diagnostic biomarker can help in the early diagnosis of cases that require emergent surgery to reduce the rates of morbidity and mortality, reducing the length of stay in the ED and lowering health care costs. The aim of this study was to evaluate the serum and urine S100A8/A9 and serum amyloid A biomarkers in patients presenting with acute abdominal pain in the ED, as a diagnostic biomarker for emergent surgery.

## 2. Methods

### 2.1. Participants

In this prospective diagnostic test accuracy (DTA) study, patients with recent abdominal pain referred to the ED of Imam Khomeini General Hospital, from January 2016 to January 2017, were enrolled. This study was approved and supported by the Jundishapur University of Medical Sciences, Ahvaz, Iran. The ethical committee of the university confirmed this study with ethical codes IR.AJUMS.REC1394.82, IR.AJUMS.REC1394.88, and IR.AJUMS.REC1394.87, and all patients signed a written informed consent prior to enrollment.

### 2.2. Inclusion Criteria

Patients over 18 years of age, presenting to the ED with recent abdominal pain (under 72 hours), with abdominal tenderness and rebound tenderness on examination are included in the study.

### 2.3. Exclusion Criteria

Patients who are pregnant with flank pain alone, altered mental status, evidence of malignancy, history of autoimmune disease, inflammatory bowel disease, history of abdominal trauma, and surgery over the past two weeks are excluded in the study.

### 2.4. Test Methods

After the admission of the patients, necessary information, including age, sex, location of the pain, onset time of the pain, associated symptoms, and early clinical diagnosis (after history taking and physical examination), was recorded. Paraclinical measures, including blood sampling for the required tests and appropriate imaging methods, were taken. In addition, for all the patients entering the study, blood samples and urine specimens were sent for the measurement of serum and urine levels of S100A8/A9 and the serum level of amyloid A. The blood samples were centrifuged in the laboratory to separate their serum, kept at a temperature of −20°C, along with urine specimens, until the estimated sample size and the ultimate measurements were assessed. All patients were referred to a general surgeon and were carefully checked on. The final diagnosis of the patients who underwent surgery was made based on the pathology report by the responsible surgeon. For patients not requiring surgery, a final probable clinical diagnosis was based on the history, physical examination, paraclinical measures, and the final opinion of the general surgeon and consultants in different specialties, according to scientific and academic standards. After the estimated sample size was reached, all of the samples were analyzed for the serum and urine levels of S100A8/A9 and the serum level of amyloid A under the supervision of a pathologist. The normal values of biomarkers were determined using the human commercial ELISA kit of S100A8/A9 and serum amyloid A (Shanghai Crystal Day Biotech Co.) (normal serum S100A8/A9 and urine S100A8/A9: 100 *μ*g/cc, normal serum amyloid A: 9.02 *μ*g/cc). Finally, the white blood cell (WBC) count and the levels of serum and urinary biomarkers, based on the final diagnosis, were measured and compared in the surgical and nonsurgical groups. In total, 246 participants, comprising two subgroups according to acute abdominal pain status, were selected. The groups were named and composed as follows: PSI+/− (PSI, pain in the surgical intervention group), comprising subjects in whom acute abdominal pain remitted postsurgically, and PNSI+/− (PNSI, pain in the nonsurgical intervention group), consisting of those subjects in whom acute abdominal pain remitted after nonsurgical intervention.

### 2.5. Statistical Analysis

The collected data was performed by descriptive statistics, such as mean, standard deviation, frequency and percentage, and analytical statistics, including the nonparametric Mann–Whitney *U* test and the Pearson correlation coefficient. The ROC curve analysis was used to evaluate the accuracy of blood cells and the serum and urine biomarkers in the diagnosis of acute abdominal pain requiring surgery. SPSS 21 software was used for the analysis. The significance level was considered to be less than 0.05.

## 3. Results

In this study, a total of 246 patients were presented to the ED with clinical findings of acute abdominal pain; of these, 66 patients were excluded and 181 patients were studied and finally analyzed. Baseline characteristics of both the study groups are given in Table 1.

**Figure 1:**
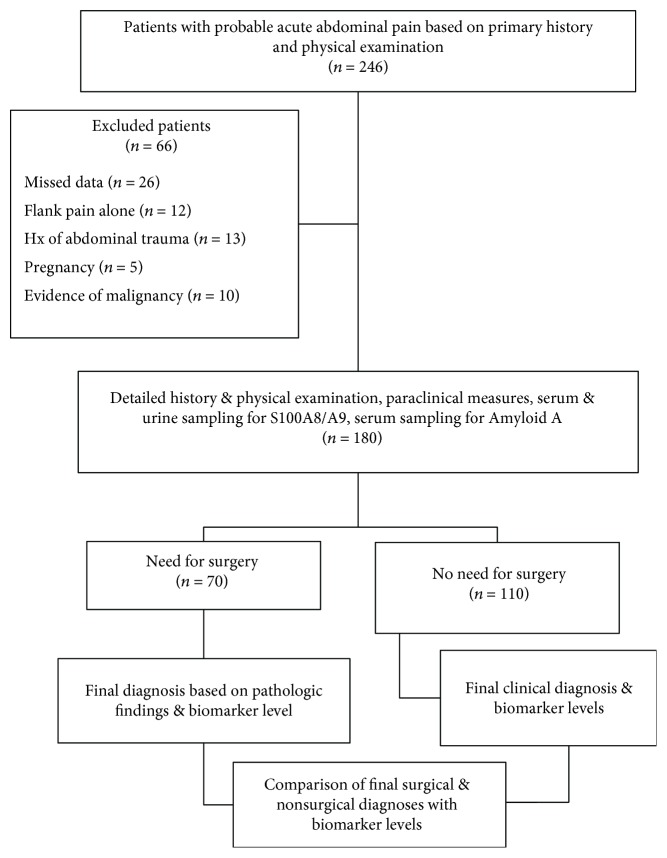
Schematic flowchart for the map of the study.

Of these, 71 patients underwent surgery (surgical group), and clinical diagnoses without the need for surgery were considered for 110 patients (nonsurgical group) (Figure 1).

The results showed that the levels of biomarkers were not statistically different between the surgical and the nonsurgical groups, in terms of mean age and gender distribution (*P* < 0.05).

In the surgical group (71 patients), the major final diagnosis included acute appendicitis (57.7%), acute cholecystitis (15.4%), and bowel obstruction (7%), leaving 19.9% with other kinds of diagnoses including complicated ovarian cyst (*n* = 2), volvulus (*n* = 2), stomach perforation (*n* = 3), ectopic pregnancy (*n* = 1), colon tubu-adenomatosis tumor (*n* = 1), mesenteric ischemia (*n* = 1), pancreas abscess (*n* = 1), complicated ovarian tumor (*n* = 1), and duodenal perforation (*n* = 2) (Table 1 of Supplementary Material).

Our findings in this group showed that the mean levels of serum in S100A8/A9 were higher than normal, in the final diagnosis, except in mesenteric ischemia. Moreover, the mean urinary levels of S100A8/A9 were higher than normal in the final diagnosis except in volvulus. The mean serum levels of amyloid A, in the final diagnosis, were higher than normal, except in intestinal volvulus and ectopic pregnancy. Finally, the mean WBC count was higher than normal, in all cases, except in bowel obstruction (Table 2).

In the nonsurgical group (110 patients), the major final clinical diagnosis included ovarian cysts (22.7%), acute pancreatitis (10.9%), dyspepsia (8.1%), acute cholecystitis (5.4%), renal colic because of stones (5.4%), and other diagnoses (21.2%): ectopic pregnancy (*n* = 3), acute cholangitis (*n* = 1), surgery site abscess (*n* = 1), gastritis (*n* = 2), acute hepatitis (*n* = 1), urinary tract infection (*n* = 2), biliary colic (*n* = 4), incarcerated periumbilical hernia (*n* = 1), varicocele (*n* = 1), pelvic inflammatory disease (*n* = 1), and mittelschmerz (*n* = 1) (Table 2 of Supplementary Material), thus leaving 26.3% with nonspecific abdominal pain (NSAP).

Our findings in this group showed that the mean serum and urine levels of S100A8/A9 were higher than normal in the final clinical diagnosis of acute pancreatitis, ectopic pregnancy, acute cholangitis, surgery site abscess, gastritis, acute hepatitis, ileus, and inflammatory pelvic disease and were normal in other diagnoses.

In all diagnoses, except renal colic because of stone, the mean serum levels of amyloid A were higher than normal. Finally, the mean WBC counts were higher than normal in patients with a final diagnosis of ovarian cysts, acute cholecystitis, acute pancreatitis, ectopic pregnancy, dyspepsia, ileus, urinary tract infection, biliary colic, and incarcerated periumbilical hernia (Table 3).

The results showed that the mean levels of serum and urine of S100A8/A9 biomarkers and WBC count were significantly higher in the surgical group than in the nonsurgical group. The results of the nonparametric Mann–Whitney *U* test showed a statistically significant difference in these biomarkers between the surgical and the nonsurgical groups.

Among common diagnoses between the two groups (ovarian cysts, acute cholecystitis, and ectopic pregnancies), the mean WBC count was significantly higher in complicated ovarian cysts in the surgical group than in the nonsurgical group (*P* < 0.05). Moreover, the mean level of serum and urine S100A8/A9 in acute cholecystitis was significantly higher in the surgical group than in the nonsurgical group (*P* < 0.05). However, there was no significant difference in the mean levels of biomarkers in ectopic pregnancy between the two groups (*P* < 0.05) (Table 4).

In our study, the data in Table 5 show the relationship between the time of onset of pain, levels of biomarkers, and WBC counts in the surgical and the nonsurgical groups.

The nonsurgical group had a statistically significant direct correlation between the mean time of onset of pain until the patient's arrival, with the serum and urine levels of S100A8/A9 and the serum amyloid A biomarkers. In other words, with increased “mean time of onset of pain until patient arrival,” the mean levels of serum and urine S100A8/A9 and serum amyloid A biomarkers will increase and vice versa.

However, in the surgical group, there was a significant inverse correlation between the “mean time of pain onset until patient arrival” and the mean levels of the serum S100A8/A9 biomarker.

A strong correlation between the relative increases of S100A8/A9 and that of WBC in PSI+/−, but not in PNSI+/−, was found. Interestingly, the correlation of the increase in S100A8/A9 and that of serum amyloid A progressed across the two groups being nonsignificant in group PNSI+/− and moderately to highly significant in group PSI+/− (Table 6).

**Figure 2:**
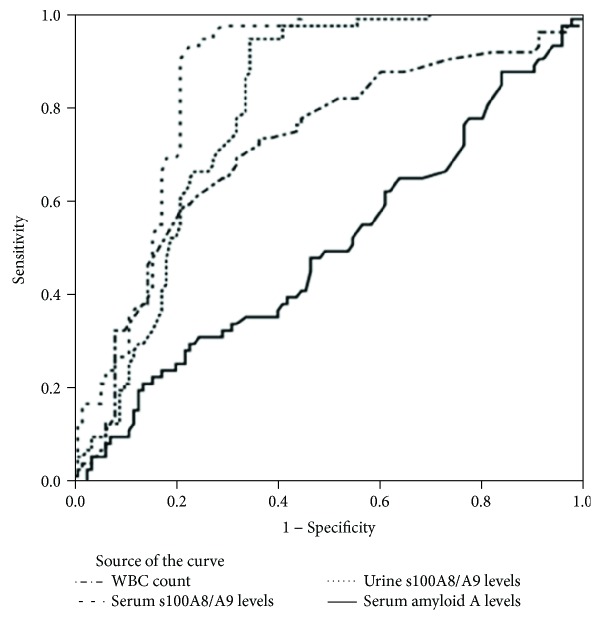
Diagnostic accuracy of white blood cells and serum and urinary biomarkers in the diagnosis of acute abdominal pain requiring surgery.

Finally, the results of the ROC curve analysis showed that in anticipation of the need for surgery in acute abdominal pain, the diagnostic accuracy of the WBC count, serum S100A8/A9, urine S100A8/A9, and serum amyloid A were 71%, 86%, 79%, and 50%, respectively (Figure 2).

The summary of diagnostic values of each biomarker in the study participants has been given in Table 7.

## 4. Discussion

The studied biomarkers are produced and secreted in response to the immune system stimulation and continuation of the inflammatory processes; therefore, the measurement of their presence and quantity in body fluids and tissues can help in the early diagnosis and prognosis of some diseases.

In the present study, in contrast to other studies that considered a specific diagnosis, we considered a wider range of patients with acute abdominal pain in terms of the final outcome and biomarkers. Our results showed that in many other diagnoses in the abdominal and pelvic areas as well as acute appendicitis, which accounts for most patients, the mean levels of the S100A8/A9 biomarker increase. In fact, it is assumed that the diagnoses mentioned are associated with infectious or inflammatory processes and immune system stimulation, which have further led to increased levels of S100A8/A9.

In recent years, S100A8/A9 was studied as a biomarker secreted in serum, urine, and feces in some infectious or inflammatory diseases of the gastrointestinal tract, like inflammatory bowel disease, familial Mediterranean fever, appendicitis, infections, bowel inflammation, and so on, especially in children. Some of these studies confirmed the role of S100A8/A9 in the diagnosis, clinical course, and prognosis of the disease [7, 31, 32, 37, 38]. Other studies have emphasized the importance of signs and symptoms of the disease and the use of other diagnostic methods and routine biomarkers [32–35].

In this study, we also showed that the levels of serum amyloid A increase in various diagnoses with activated inflammatory or immune responses.

Mayer et al. showed that serum amyloid A can predict the severity of the disease better than CRP, in acute pancreatitis [39]. In addition, Sit et al. showed that although clinical signs and symptoms play a great role in the diagnosis of acute appendicitis, the measurement of serum amyloid A can help surgeons avoid unnecessary laparotomy [18].

The results of our study showed that the mean levels of serum and urine of S100A8/A9 biomarkers and the WBC count in the surgical group were considerably more than those in the nonsurgical group and can prove to be useful in anticipating the need for surgery in patients with acute abdominal pain, along with other factors. However, serum amyloid A does not seem helpful in this regard.

The heterogeneities in the surgical and the nonsurgical groups, among some diagnoses with inflammatory natures and the levels of biomarkers, may be due to the difference in the time of measurement of the samples, including the onset of the inflammatory process, confounding factors, and technical problems associated with their measurement.

In our study, some diagnoses were common in the surgical and the nonsurgical groups when levels of biomarkers where compared.

In “abdominal pain caused by complicated ovarian cysts,” the mean level of serum and urine of S100A8/A9 and the WBC count were higher than normal in the surgical group. But in the nonsurgical group, only the WBC count and serum amyloid A were higher. Finally, it can be stated that regarding the statistical significance, only the WBC count can predict the need for surgery in complicated ovarian cyst.

In the diagnosis of “abdominal pain caused by acute cholecystitis,” the mean level of serum and urine of S100A8/A9, the WBC count, and serum amyloid A were higher than normal in the surgical group and only the WBC count and serum amyloid were higher than normal in the nonsurgical group. Statistically, it seems that only the serum and urine levels of the S100A8/A9 biomarker can predict the need for surgery in acute cholecystitis.

In the diagnosis of “abdominal pain caused by ectopic pregnancy,” the mean level of serum and urine of S100A8/A9 and the WBC count were higher than normal in both the surgical and the nonsurgical groups. However, the mean level of serum amyloid A was higher only in the nonsurgical group. Statistically, none of these indicators can be useful in predicting the need for surgery in ectopic pregnancy.

Finally, the results of this study showed that the highest diagnostic accuracy for surgery requirement was related to the serum S100A8/A9 biomarker, followed by the urine S100A8/A9 biomarker. Serum amyloid A biomarker had a low and nonsignificant diagnostic accuracy that may be valuable in combination with other biomarkers.

The results of our study showed that in the nonsurgical group, the levels of biomarkers had a weak positive association with the mean time of pain onset until the arrival of the patient. In other words, the longer the onset of the patient's pain until arrival, the greater the levels of biomarkers.

But among the patients in the surgical group, the levels of biomarkers had a weak negative association with the onset of the patient's pain until arrival, which means that the longer the onset of the patient's pain until arrival, the lower the levels of biomarkers.

In the nonsurgical group, only the WBC count had a nonsignificant positive association, but in the surgical group, serum S100A8/A9 alone had a significantly inverse relationship (Table 5).

Assessment of the mean time of onset of pain among patients until arrival in both the surgical and the nonsurgical groups and the time of the measurement of the biomarkers resulted in different results that cannot easily be justified.

Our findings showed that S100A8/A9 but not amyloid A is increased in the surgical group. Though we cannot prove a causal relation for these findings, the possible reason is that amyloid A is an acute-phase reactant that is produced in the liver but, in patients of the surgical group with mentioned specific diagnoses, could not stimulate the liver for enough production. More studies are needed for clarification.

Mills et al. [7] investigated the role of S100A8/A9 biomarkers in patients with acute lower abdominal pain and found that the problems of purchase, transfer of the kits, the shipping effect, and the delay in sample analysis of the biomarkers increase the sensitivity and decrease the specificity and the diagnostic accuracy of the test.

## 5. Limitations

The heterogeneous composition of the two groups with certain diagnoses in both groups and the low number of cases limit the validity of our study. Also, in our study, due to the problems associated with the purchase and shipment of the kits, the costs, and the technical limitations of measurement, sampling was performed at the baseline. After reaching the sample size, the levels of biomarkers were simultaneously measured. Logically, any delay in the analysis of the sample should, perhaps, lead to increased levels of biomarkers, due to cell lysis and the release of the cellular contents. Also, it seems pharmacokinetic and pharmacodynamic characteristics of biomarkers and inflammation severity of patients affect the biomarker levels in correlation with the onset of pain times. We could not explain these differences and discrepancies in the levels of biomarkers with respect to the pain onset and admission time.

## 6. Conclusion

Though our data may show that the serum and urine S100A8/A9 can be useful indicators of the need for surgery in patients presenting with acute abdominal pain at the emergency department, the data rather suggest that patients in the surgical group were more seriously ill than those in the nonsurgical group, with a correspondingly higher risk of operation. Serum amyloid A had a low and nonsignificant diagnostic accuracy, which may be valuable in combination with clinical findings and other biomarkers.

## Figures and Tables

**Table 1:** Comparing baseline characteristics between the two groups of the study participants.

Characteristics	Surgical (*n* = 71)	Nonsurgical (*n* = 110)	*P* values
Age (years)^∗^	37.04 ± 15.86	34.81 ± 14.4	0.102
Gender^∗∗^			
Male	31 (43.7%)	44 (40%)	0.965
Female	25 (40.3%)	66 (60%)	0.0541
WBC count (×10^9^/L)^∗^	12.7 ± 10.14	9.82 ± 3.68	0.0445
Serum S100A8/A9^∗^	208.17 ± 101.5	95.95 ± 84.3	0.0238
Urine S100A8/A9^∗^	147.64 ± 44.5	81.22 ± 64.4	0.0315
Serum amyloid A^∗^	21.37 ± 20.38	20.95 ± 23.09	0.996
Pain onset			
Admission day	20 (28.2%)	31 (28.2%)	1.00
<5 days before admission	42 (59.2%)	63 (57.3%)	0.998
5–10 days before admission	8 (11.2%)	13 (11.8%)	1.00
>10 days before admission	1 (1.4%)	3 (2.7%)	0.985

^∗^Mean ± SD. ^∗∗^Number (%)

**Table 2:** Distribution of mean white blood cell count and levels of biomarkers in different diagnoses based on pathological findings in the surgical group (in patients in which their numbers were ≥5).

Final diagnosis	Statistics	WBC count	Serum S100A8/A9	Urine S100A8/A9	Serum amyloid A
Acute appendicitis *N* = 41	Mean	11334.15	218.19	153.39	22.73
	Std. deviation	3237.02	98.33	44.99	21.50
	Minimum	3200.0	75.0	79.0	6.70
	Maximum	20000.0	518.0	263.0	84.70

Acute cholecystitis *N* = 11	Mean	19318.18	244.91	140.73	17.75
	Std. deviation	24309.09	144.94	20.11	11.99
	Minimum	10000.0	138.0	110.0	7.60
	Maximum	92500.0	545.0	169.0	40.60

Gastrointestinal obstruction *N* = 5	Mean	8980.0	173.80	121.20	9.62
	Std. deviation	2534.17	61.63	45.53	3.09
	Minimum	6200.0	92.0	47.00	5.50
	Maximum	13000.0	260.0	155.0	12.80

**Table 3:** Distribution of mean white blood cell count and levels of biomarkers in different diagnoses based on clinical diagnosis and paraclinical tests in the nonsurgical group (in patients in which their numbers were ≥5).

Final diagnosis	Statistics	WBC Count	Serum S100A8/A9	Urine S100A8/A9	Serum amyloid A
Complicated ovarian cyst *N* = 25	Mean	9216.0	85.28	77.0	16.88
	Std. deviation	1640.80	68.79	61.99	17.03
	Minimum	6000.0	19.0	8.0	4.60
	Maximum	12000.0	282.0	229.0	78.00

Acute cholecystitis *N* = 6	Mean	12400.0	70.0	49.33	17.53
	Std. deviation	3384.67	61.02	41.62	11.07
	Minimum	7400.00	29.0	13.0	7.70
	Maximum	18000.00	189.0	125.0	39.30

Acute pancreatitis *N* = 12	Mean	13225.00	187.42	134.42	32.95
	Std. deviation	7097.65	142.86	45.24	35.72
	Minimum	5500.05	50.0	48.0	7.80
	Maximum	33800.05	559.0	238.0	129.0

Renal colic *N* = 6	Mean	7000.0	60.17	47.33	8.57
	Std. deviation	1454.65	27.88	26.49	3.72
	Minimum	4500.0	23.0	16.0	4.80
	Maximum	8200.0	94.0	94.0	15.70

Dyspepsia *N* = 9	Mean	10122.22	43.45	30.44	22.98
	Std. deviation	1856.60	26.06	20.21	22.89
	Minimum	7800.0	23.0	9.0	4.80
	Maximum	13300.0	91.0	77.0	71.40

Ileus *N* = 5	Mean	9060.0	106.400	104.2000	21.8400
	Std. deviation	2822.76	69.6046	42.67552	26.92142
	Minimum	4800.0	50.0	51.00	7.80
	Maximum	12000.0	208.0	154.00	69.70

Nonspecific abdominal pain *N* = 29	Mean	8724.14	79.83	59.35	17.15
	Std. deviation	3032.92	64.75	59.64	17.57
	Minimum	5200.0	12.0	9.00	6.50
	Maximum	21000.0	314.0	213.0	93.0

**Table 4:** Distribution of mean white blood cell count and levels of biomarkers in common diagnoses based on the surgical and the nonsurgical groups.

Common diagnosis	Group	Statistics	WBC count	Serum S100A8/A9	Urine S100A8/A9	Serum amyloid A
Complicated ovarian cyst	Without surgery *N* = 25	Mean	9216.0	85.28	77.0	16.88
		Std. deviation	1640.80	68.79	61.99	17.03
		Minimum	6000.0	19.0	8.0	4.60
		Maximum	12000.0	282.0	229.0	78.0
	Surgery *N* = 2	Mean	12500.0	185.0	169.0	3.55
		Std. deviation	424.26	59.40	84.85	4.46
		Minimum	12200.0	143.0	109.0	0.40
		Maximum	12800.0	227.0	229.0	6.70
		*P*-value^¥^	0.006^∗^	0.07	0.09	0.50

Acute cholecystitis	Without surgery *N* = 6	Mean	12400.0	70.0	49.33	17.53
		Std. deviation	3384.67	61.02	41.62	11.07
		Minimum	7400.0	29.0	13.00	7.70
		Maximum	18000.0	189.0	125.00	39.30
	Surgery *N* = 11	Mean	19318.12	244.91	140.73	17.75
		Std. deviation	24309.09	144.94	20.11	11.99
		Minimum	10000.0	138.0	110.0	7.60
		Maximum	92500.0	545.0	169.0	40.60
		*P* value^¥^	0.99	0.005^∗^	0.001^∗^	0.88

Ectopic pregnancy	Without surgery *N* = 3	Mean	13566.67	149.0	150.33	33.20
		Std. deviation	4377.60	87.02	54.86	22.23
		Minimum	9500.0	63.0	111.0	9.70
		Maximum	18200.0	237.0	213.0	53.90
	Surgery *N* = 1	Mean	16000.0000	336.000	251.0000	7.9000
		*P* value^¥^	0.990	0.50	0.50	0.50

^∗^Statistically significant *P* value. ^¥^ *P* value from the Mann–Whitney *U* test.

**Table 5:** Distribution of the mean time of onset of pain until patient referral on the levels of white blood cell count and biomarker between the surgical and the nonsurgical groups.

Group	Variable	Correlation/*P* value^¥^	Time
Without surgery *N* = 110	WBC count	Correlation coefficient	0.130
		*P* value	0.175
	Serum S100A8/A9	Correlation coefficient	0.225
		*P* value	0.018^∗^
	Urine S100A8/A9	Correlation coefficient	0.274
		*P* value	0.004^∗^
	Serum amyloid A	Correlation coefficient	0.270
		*P* value	0.004^∗^

Surgery *N* = 71	WBC count	Correlation coefficient	−0.028
		*P* value	0.816
	Serum S100A8/A9	Correlation coefficient	−0.253
		*P* value	0.034^∗^
	Urine S100A8/A9	Correlation coefficient	−0.193
		*P* value	0.106
	Serum amyloid A	Correlation coefficient	−0.116
		*P* value	0.335

^∗^Statistically significant *P* value. ^¥^ *P* value from the Mann–Whitney *U* test.

**Table 6:** Spearman rank correlation coefficients of the relative increase of S100A8/A9 to those of WBC and serum amyloid A.

Variables	PSI+/−			PNSI+/−		
	*N*	*r*	*P* value	*N*	*R*	*P* value
WBC	52	0.901	0.002	23	0.432	0.060
Serum amyloid A	44	0.58	0.01	30	0.438	0.064

PSI+/− (PSI, pain in the surgical intervention group), comprising subjects in whom acute abdominal pain remitted postsurgically, and PNSI+/− (PNSI, pain in the nonsurgical intervention group), consisting of those subjects who were acute abdominal pain remitted after nonsurgical intervention.

**Table 7:** Summary of diagnostic accuracy of various biomarkers of interest in the study group.

Test result variable(s)	AUC	SE	Sensitivity	Specificity	*P* value	Asymptotic 95% confidence interval	
						Lower bound	Upper bound
WBC count (×10^9^/L)	0.713	0.040	97.5%	76%	<0.001	0.634	0.792
Serum S100A8/A9	0.855	0.028	91.5%	87.2%	<0.001	0.799	0.911
Urine S100A8/A9	0.791	0.033	93%	77.5%	<0.001	0.725	0.856
Serum amyloid A	0.499	0.045	33.6%	43.5%	.984	0.412	0.587

## Supplementary Materials

Supplementary Materials Supplementary material included two tables. They show the distribution of mean white blood cell count and the levels of biomarkers in different diagnoses based on pathological findings in the surgical and the nonsurgical groups in patients in which their numbers were less than 5. In the surgical group (Table 1 of Supplementary Material), some diagnoses were less than 5 in number, in which biomarker levels were calculated and recorded for them and used for the final analysis. They included complicated ovarian cyst, volvulus, stomach perforation, ectopic pregnancy, colon tubu-adenomatosis tumor, mesenteric ischemia, pancreas abscess, complicated ovarian tumor, and duodenal perforation. Also, in the nonsurgical group (Table 2 of Supplementary Material), some diagnoses were less than 5 in number, in which biomarker levels were calculated and recorded for them and used for the final analysis. They included ectopic pregnancy, acute cholangitis, surgery site abscess, gastritis, acute hepatitis, urinary tract infection, biliary colic, incarcerated periumbilical hernia, varicocele, pelvic inflammatory disease, and mittelschmerz.
