# Supplementary material for: Diagnostic Accuracy of Serum and Urine S100A8/A9 and Serum Amyloid A in Probable Acute Abdominal Pain at Emergency Department
Source: Dis Markers. 2018 Jul 3;2018:6457347. doi: 10.1155/2018/6457347 (PMC6051260; doi:10.1155/2018/6457347)
Supplement: Supplementary Materials — Supplementary material included two tables. They show the distribution of mean white blood cell count and the levels of biomarkers in different diagnoses based on pathological findings in the surgical and the nonsurgical groups in patients in which their numbers were less than 5. In the surgical group (Table 1 of Supplementary Material), some diagnoses were less than 5 in number, in which biomarker levels were calculated and recorded for them and used for the final analysis. They included complicated ovarian cyst, volvulus, stomach perforation, ectopic pregnancy, colon tubu-adenomatosis tumor, mesenteric ischemia, pancreas abscess, complicated ovarian tumor, and duodenal perforation. Also, in the nonsurgical group (Table 2 of Supplementary Material), some diagnoses were less than 5 in number, in which biomarker levels were calculated and recorded for them and used for the final analysis. They included ectopic pregnancy, acute cholangitis, surgery site abscess, gastritis, acute hepatitis, urinary tract infection, biliary colic, incarcerated periumbilical hernia, varicocele, pelvic inflammatory disease, and mittelschmerz. [file 6457347.f1.docx]

***Table1*: Distribution of mean white blood cell count and the levels of biomarkers in different diagnoses based on pathological findings in the surgical group (in patients which their numbers were<5)**

| **Serum Amyloid A** | **Urine S100A8/A9** | **Serum S100A8/A9** | **WBC Count** | **Statistics** | **Final Diagnosis** |
| --- | --- | --- | --- | --- | --- |
| 3.55 | 169.0 | 185.0 | 12500.0 | Mean | **Complicated Ovarian Cyst N=2** |
| 4.46 | 84.85 | 59.40 | 424.26 | Std. Deviation |  |
| 0.40 | 109.0 | 143.0 | 12200.0 | Minimum |  |
| 6.70 | 229.00 | 227.0 | 12800.0 | Maximum |  |
| 7.65 | 84.0 | 120.0 | 13750.0 | Mean | **Volvulus N=2** |
| 0.50 | 70.71 | 38.18 | 1767.77 | Std. Deviation |  |
| 7.30 | 34.00 | 93.0 | 12500.0 | Minimum |  |
| 8.00 | 134.00 | 147.0 | 15000.0 | Maximum |  |
| 22.70 | 119.0 | 146.67 | 10100.0 | Mean | **Stomach Perforation N=3** |
| 25.41 | 14.0 | 23.72 | 5273.52 | Std. Deviation |  |
| 6.70 | 109.0 | 125.0 | 4100.0 | Minimum |  |
| 52.0 | 135.0 | 172.0 | 14000.0 | Maximum |  |
| 7.90 | 251.0 | 336.0 | 16000.0 |  | **Ectopic Pregnancy N=1** |
| 11.0 | 219.0 | 146.0 | 12000.0 |  | **Colon Tuboloadenomatous Tumor N=1** |
| 64.80 | 150.0 | 71.0 | 15000.0 |  | **Mesentric Ischemia N=1** |
| 16.30 | 145.0 | 156.0 | 13700.0 |  | **Pancreas Abcess N=1** |
| 47.30 | 139.0 | 130.0 | 9000.0 |  | **Complicated Benign Ovarian Tumor N=1** |
| 52.15 | 136.50 | 191.50 | 18000.0 | Mean | **Duedenal Perforation**  **N=2** |
| 33.30 | 16.26 | 45.96 | 7071.07 | Std. Deviation |  |
| 28.60 | 125.0 | 159.0 | 13000.0 | Minimum |  |
| 75.70 | 148.00 | 224.0 | 23000.0 | Maximum |  |

In surgical group, some diagnoses were less than 5 in number ,which biomarker levels calculated and recorded for them and used for final analysis. They included; complicated ovarian cyst (N=2), volvulus (N=2), stomach perforation (N=3), ectopic pregnancy (N=1) ,colon tubuadenomatosis tumor (N=1), mesenteric ischemia (N=1), pancreas abcess (N=1), complicated ovarian tumor (N=1) and duedenal perforation (N=2).

***Table2*: Distribution of mean white blood cell count and the levels of biomarkers in different diagnoses based on clinical diagnosis and paraclinical tests in the nonsurgical group.**

| **Serum Amyloid A** | **Urine S100A8/A9** | **Serum S100A8/A9** | **WBC Count** | **Statistics** | **Final Diagnosis** |
| --- | --- | --- | --- | --- | --- |
| 33.20 | 150.33 | 149.0 | 13566.67 | Mean | **Ectopic Pregnancy N=3** |
| 22.23 | 54.86 | 87.02 | 4377.60 | Std. Deviation |  |
| 9.70 | 111.0 | 63.0 | 9500.0 | Minimum |  |
| 53.90 | 213.0 | 237.0 | 18200.0 | Maximum |  |
| 13.0 | 116.0 | 209.0 | 9000.0 |  | **Acute Chollangitis N=1** |
| 29.80 | 205.0 | 234.0 | 5900.0 | Mean | **Surgery Site Abcess N=1** |
| 10.9000 | 157.5000 | 104.500 | 8000.0 | Mean | **Gastritis N=2** |
| 3.67696 | 27.57716 | 62.9325 | 282.84 | Std. Deviation |  |
| 8.30 | 138.00 | 60.0 | 7800.0 | Minimum |  |
| 13.50 | 177.00 | 149.0 | 8200.0 | Maximum |  |
| 8.9000 | 222.0000 | 280.000 | 8000.0 |  | **Acute Hepatitis N=1** |
| 31.6500 | 77.5000 | 68.500 | 9500.0 | Mean | **Urinary Tract Infection**  **N=2** |
| 27.93072 | 71.41778 | 12.0208 | 0.00 | Std. Deviation |  |
| 11.90 | 27.00 | 60.0 | 9500.0 | Minimum |  |
| 51.40 | 128.00 | 77.0 | 9500.0 | Maximum |  |
| 18.78 | 76.25 | 54.75 | 11775.0 | Mean | **Billiary Colic**  **N=4** |
| 7.86 | 59.34 | 12.15 | 3425.76 | Std. Deviation |  |
| 9.00 | 21.0 | 38.0 | 8500.0 | Minimum |  |
| 27.00 | 131.0 | 67.0 | 16600.0 | Maximum |  |
| 138.0 | 238.0 | 79.0 | 15700.0 | Mean | **Incarcerated periumblical Hernia**  **N=1** |
| 28.70 | 43.0 | 60.0 | 8000.0 |  | **Varicocele N=1** |
| 29.80 | 209.0 | 234.0 | 9000.0 |  | **Pelvic Inflammatory Disease N=1** |
| 9.7 | 42.0 | 47.0 | 7000.0 |  | **Mitel Schmers N=1** |

In nonsurgical group, some diagnoses were less than 5 in number ,which biomarker levels calculated and recorded for them and used for final analysis. They included; ectopic pregnancy (N=3), acute cholangitis (N=1), surgery site abcess (N=1), gastritis (N=2), acute hepatitis (N=1), urinary tract infection (N=2), biliary colic, (N=4) incarcerated periumblical hernia (N=1), varicocele (N=1), pelvic inflammatory disease (N=1) , mitel schmers (N=1).
